# Treatment with macrolides and glucocorticosteroids in severe community-acquired pneumonia: A post-hoc exploratory analysis of a randomized controlled trial

**DOI:** 10.1371/journal.pone.0178022

**Published:** 2017-06-15

**Authors:** Adrian Ceccato, Catia Cilloniz, Otavio T. Ranzani, Rosario Menendez, Carles Agusti, Albert Gabarrus, Miquel Ferrer, Oriol Sibila, Michael S. Niederman, Antoni Torres

**Affiliations:** 1 Department of Pneumology, Institut Clinic de Respiratori, Hospital Clinic of Barcelona—Institut d'Investigacions Biomèdiques August Pi i Sunyer (IDIBAPS), University of Barcelona (UB)—SGR 911—Ciber de Enfermedades Respiratorias (Ciberes, CB06/06/0028) Villarroel, Barcelona, Spain; 2 Seccion Neumologia, Hospital Nacional Prof. Alejandro Posadas, Illia y Marconi s/n Palomar, Argentina; 3 Respiratory Intensive Care Unit, Pulmonary Division, Heart Institute, Hospital das Clínicas, University of São Paulo, Av. Dr. Arnaldo, 455-Cerqueira César—CEP: São Paulo, Brazil; 4 Servicio de Neumología, IIS/Hospital Universitario y Politécnico La Fe, Avinguda de Fernando Abril Martorell, Valencia, CIBERES, Spain; 5 Servei de Pneumologia, Hospital de la Santa Creu i Sant Pau, Sant Antoni Maria Claret 167, Barcelona, Spain; 6 Division of Pulmonary and Critical Care Medicine, Weill Cornell Medical College, New York Presbyterian/Weill Cornell Medical Center, NY, New York, NY, United States of America; Hospital Sao Francisco Xavier, PORTUGAL

## Abstract

**Background:**

Systemic corticosteroids have anti-inflammatory effects, whereas macrolides also have immunomodulatory activity in addition to their primary antimicrobial actions. We aimed to evaluate the potential interaction effect between corticosteroids and macrolides on the systemic inflammatory response in patients with severe community-acquired pneumonia to determine if combining these two immunomodulating agents was harmful, or possibly beneficial.

**Methods:**

We performed a post-hoc exploratory analysis of a randomized clinical trial conducted in three tertiary hospitals in Spain. This trial included patients with severe community-acquired pneumonia with high inflammatory response (C-reactive protein [CRP] >15 mg/dL) who were randomized to receive methylprednisolone 0.5 mg/kg/tpd or placebo. The choice of antibiotic treatment was at the physician's discretion. One hundred and six patients were classified into four groups according to antimicrobial therapy combination (β-lactam plus macrolide or β-lactam plus fluoroquinolone) and corticosteroid arm (placebo or corticosteroids). The primary outcome was treatment failure (composite outcome of early treatment failure, or of late treatment failure, or of both early and late treatment failure).

**Results:**

The methylprednisolone with β-lactam plus macrolide group had more elderly patients, with comorbidities, and higher pneumonia severity index (PSI) risk class V, but a lower proportion of intensive care unit admission, compared to the other groups. We found non differences in treatment failure between groups (overall p = 0.374); however, a significant difference in late treatment failure was observed (4 patients in the placebo with β-lactam plus macrolide group (31%) vs. 9 patients in the placebo with β-lactam plus fluoroquinolone group (24%) vs. 0 patients in the methylprednisolone with β-lactam plus macrolide group (0%) vs. 2 patients [5%] in the methylprednisolone with β-lactam plus fluoroquinolone group overall p = 0.009). We found a significant difference for In-hospital mortality in the per protocol population (overall p = 0.01). We did not find significant differences in treatment failure, early or late; or In-hospital mortality after adjusting for severity (PSI), year and centre of enrolment.

**Conclusions:**

In this exploratory analysis, we observed that the glucocorticosteroids and macrolides combination had no statistically significant association with main clinical outcomes compared with other combinations in patients with severe community acquired pneumonia and a high inflammatory response after taking account potential confounders.

**Trial registration:**

Clinicaltrials.gov NCT00908713.

## Background

Despite advances in antibiotic treatment, the mortality of hospitalized community-acquired pneumonia (CAP) patients is still high, especially in those with severe illness[1,2]. In severe CAP, high levels of inflammatory cytokines can be harmful and cause pulmonary dysfunction associated with adverse outcomes[3,4]. On the other hand, a reduced inflammatory reaction, as seen in immunosuppressed patients or elderly can be dangerous and lead to worse outcomes[5–7]. Several treatments have been tested to control the dysregulated inflammatory response in CAP [8,9], however it is still not clear which therapies could best achieve these goals, leading to improved outcomes or which could worsen the condition.

The mortality of patients with severe CAP can be high regardless of whether they receive adequate and prompt antibiotic treatment. Macrolide combination therapy was associated with reduced mortality in retrospective analyses and in non-interventional studies, mainly in patients with severe pneumonia[10,11]. The use of systemic corticosteroids in addition to the antibiotic treatment in CAP seems to have beneficial effects, mainly for severe CAP[12–16]. Systemic corticosteroids have anti-inflammatory effects and macrolides also have immunomodulatory activity in addition to their primary antimicrobial actions. Macrolides are concentrated in phagocytic cells and dampen the inflammatory response by inhibiting the generation of proinflammatory cytokines[17]. They have been used in low doses for long-term therapy in asthma[18], chronic obstructive pulmonary disease[19], cystic fibrosis[20] and bronchiectasis[21] because of these anti-inflammatory actions.

There is a gap in knowledge about whether the potential anti-inflammatory effect of corticosteroids could be potentiated by the administration of a macrolide. The combination of macrolide plus corticosteroids is currently used without a scientific evaluation, although we do not know whether this combination could decrease the inflammatory response to a very low level, increasing the risk of side effects.

We hypothesized that when macrolides are used with adjunctive glucocorticosteroid treatment, there would be a better modulation of the inflammatory response without greater risk of side effects, than with either agent alone, which could, in turn, lead to a lower rate of treatment failure.

## Methods

We conducted an exploratory post-hoc analysis of data from a multicentre, randomized, double-blind, placebo-controlled trial (Clinicaltrials.gov Identifier: NCT00908713) involving patients with both severe CAP and a high inflammatory response, defined by a level of C-reactive protein (CRP) >15 mg/dL on admission, as described in detail elsewhere[12]. We included 106 patients with severe CAP and a high inflammatory response, who received placebo or glucocorticosteroids and antibiotic therapy with a β-lactam plus macrolide or β-lactam plus fluoroquinolone. Fourteen patients who received antibiotic monotherapy were excluded from the analysis since this is not the recommended empiric antibiotic treatment for severe pneumonia. Patients with influenza infection were also excluded from the original trial.

Patients were recruited from the Departments of Pneumology of three tertiary Spanish hospitals. The local ethic committee ¨Comité Ético de Investigación Clínica del Hospital Clinic de Barcelona¨ approved the study protocol and written informed consent was obtained from all participants or from their authorized representatives.

### Antimicrobial treatment

All patients were treated with antibiotics according to current international guidelines[22]. All patients in this analysis received a β-lactam plus a macrolide or a fluoroquinolone. Switch from intravenous to oral therapy and duration of the antibiotic treatment was entirely left to the discretion of the medical team, as was the decision to transfer patients to the intensive care unit or for hospital discharge.

### Data collection

The following data were collected on admission: age, gender, smoking history, clinical symptoms, physical examination and comorbidities. The initial risk class was calculated using a pneumonia severity index (PSI) score[23] and severity criteria were assessed according to the ATS criteria modified by Ewig *et al*[24]. Patients were evaluated daily for treatment failure and time to clinical stability until discharge day [25]. Adverse events and mortality were recorded during the hospital stay.

Microbiologic examination methods are described elsewhere [12].

### Biormarker measurements

Samples for cytokine, procalcitonin and C-reactive protein (CRP) determinations were obtained on the first day and after 72h and 7 days of treatment, centrifuged and frozen at -80°C until analysis. Determination of interleukin (IL)- 6, IL-8 and IL-10 levels was performed using a commercial enzyme immunoassay technique (Biosource, Nivelles, Belgium). An immunoluminometric technique was used to measure procalcitonin (Liaison Brahms PCT; DiaSorin, Saluggia, Italy) with a detection limit of 0.3 ng/ml. CRP was measured with an immunoturbidimetric method using a commercially available test (Bayer Diagnostics, Leverkusen, Germany) with an Advia 2400.

### Outcomes

The primary outcome was the rate of treatment failure, which includes treatment failure that occurred early, late, or at both times. Secondary outcomes were the levels in inflammatory markers (IL-6, IL-8, IL-10, procalcitonin, and CRP) after 3 days of treatment, time to clinical stability, length of intensive care unit (ICU) and hospital stay, and in-hospital mortality. Early treatment failure was defined as clinical deterioration within 72 hours of treatment (including development of shock, need for invasive mechanical ventilation not present at baseline, or death). Late treatment failure was defined as radiographic progression, persistence of severe respiratory failure, development of shock, need for invasive mechanical ventilation not present at baseline, or death between 72 hours and 120 hours after treatment initiation.

### Statistical analysis

Efficacy data were analyzed for both the intention-to-treat and the per-protocol populations. The intention-to-treat population included all randomized patients who received at least one dose of the study drug. The per-protocol population included all randomized patients who met all inclusion criteria, received at least six doses of the study drug, and did not deviate substantially from the protocol.

We report the number and percentage of patients for categorical variables, the mean (standard deviation) and median (interquartile range) for continuous variables. Categorical variables were compared using the χ^2^ test or the Fisher exact test. Continuous variables were compared using nonparametric Kruskal-Wallis test. Pairwise comparisons were carried out via the Bonferroni method in order to control for the experiment-wise error rate. We also performed logistic regression models to examine differences in the rate of treatment failure, early and late treatment failure and in-hospital mortality between groups, adjusting for the PSI risk class, year of recruitment, and centre. Time to clinical stability and length of ICU and hospital stay in the groups were analyzed by means of Cox proportional hazards models, adjusting for the PSI risk class, year of recruitment, and centre. The odds ratio (OR) or hazard ratio (HR) and their 95% confidence intervals (CI) were estimated. We fitted analysis of covariance (ANCOVA) models to analyze the inflammatory response at day 3, adjusting for the baseline inflammatory marker value, PSI risk class, year of recruitment, and centre. Inflammatory markers were log-transformed to fit the ANCOVA model. Each treatment effect was estimated by the least squares mean and its 95% CI. We conducted a sensitivity analysis adjusting the previous multivariate models for age, ICU admission, year and centre of enrolment. The quality of the logistic regression models and ANCOVA models were tested using the Hosmer-Lemeshow test and Akaike information criterion, respectively. All tests were 2-tailed and significance was set at 0.05. All analyses were performed with IBM SPSS Statistics version 22.0 (Armonk, New York, USA).

## Results

**Fig 1:**
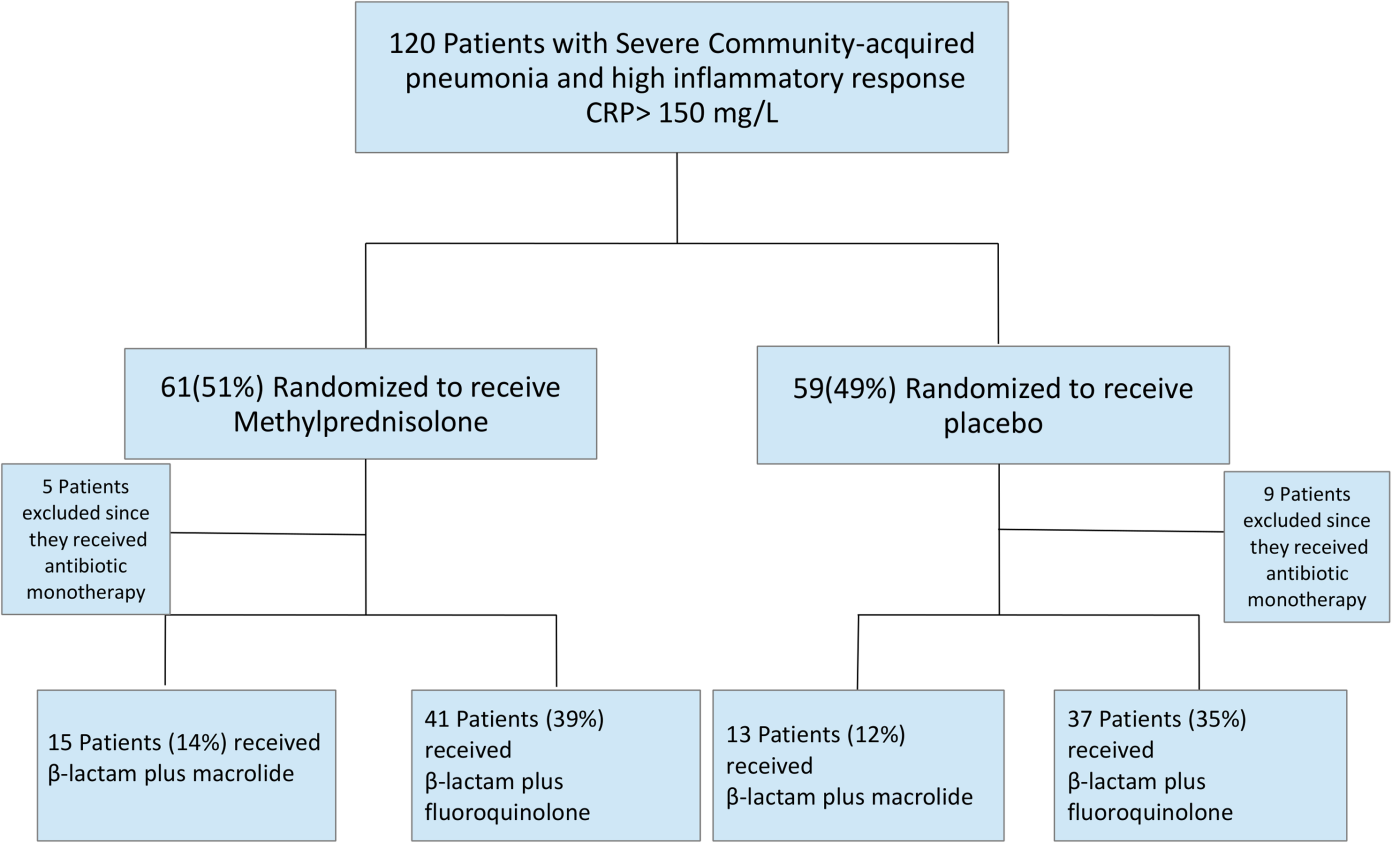
Flow diagram of the study.

Of the 106 patients, 28 received combination therapy consisting of a β-lactam plus macrolide (13 [12%] in the placebo group and 15 [14%] in the methylprednisolone group) and 78 received the combination of a β-lactam plus fluoroquinolone (37 [35%] in the placebo group and 41 [39%] in the methylprednisolone group) ¨Fig 1¨. The baseline characteristics are presented in Table 1. The methylprednisolone with β-lactam plus macrolide group had more elderly patients, with comorbidities, and higher PSI risk class V, but a lower proportion of ICU admission, compared to the other groups. The rate of etiologic diagnosis was higher in the methylprednisolone with β-lactam plus fluoroquinolone group. *Streptococcus pneumoniae* was the most common etiologic agent in the four groups (Table 2). Distribution of the pathogens did not differ among groups, except for polymicrobial infection (24% in the methylprednisolone with β-lactam plus fluoroquinolone group vs. 0% in the methylprednisone with β-lactam plus macrolide group [p = 0.018]).

**Table 1:** Baseline characteristics.

Variable	Placebo with β-lactams plus Macrolides Group (n = 13)	Placebo with β-lactams plus Fluoroquinolones Group (n = 37)	Methylprednisolone with β-lactams plus Macrolides Group (n = 15)	Methylprednisolone with β-lactams plus Fluoroquinolones Group (n = 41)	P value*
**Age, years**					**<0.001**
**Mean (SD)**	**71.1 (16.9)**	**60.5 (20.9)**	**78.5 (10.7)**	**57.2 (18.5)**	
**Median (IQR)**	**77.0 (59.0; 85.0)**	**63.0 (44.0; 77.0)c**	**82.0 (78.0; 84.0)b,d**	**58.0 (45.0; 74.0)c**	
**Male sex, No. (%)**	**10 (77)**	**22 (59)**	**8 (53)**	**23 (56)**	**0.558**
**Current smoker, No. (%)**	**3 (23)**	**13 (35)**	**0 (0)**	**14 (34)**	**0.056**
**Pre-existing comorbid conditions, No. (%)e**					
**Diabetes mellitus**	**3 (23)**	**9 (24)**	**5 (33)**	**3 (7)**	**0.090**
**Chronic pulmonary disease**	**2 (15)**	**7 (19)**	**2 (13)**	**5 (12)**	**0.867**
**Congestive heart failure**	**4 (31)**	**15 (41)**	**11 (73)d**	**10 (24)c**	**0.009**
**History of malignancy**	**2 (15)**	**5 (14)**	**1 (7)**	**0 (0)**	**0.094**
**Ischemic heart disease**	**3 (23)**	**4 (11)c**	**7 (47)b,d**	**4 (10)c**	**0.007**
**Pneumonia Severity Index score**					**<0.001**
**Mean (SD)**	**131.0 (21.7)**	**97.8 (34.0)**	**133.5 (35.5)**	**91.1 (30.1)**	
**Median (IQR)**	**132.5 (112.5; 144.5)b,d**	**104.0 (75.5; 123.5)a,c**	**134.0 (130.0; 149.0)b,d**	**94.5 (75.0; 112.5)a,c**	
**Risk class, No. (%)f**					**<0.001**
**I-III**	**0 (0)d**	**13 (35)c**	**1 (7)b**	**17 (41)a**	**0.006**
**IV**	**7 (54)**	**17 (46)**	**3 (20)**	**18 (44)**	**0.265**
**V**	**6 (46)**	**7 (19)c**	**11 (73)b,d**	**6 (15)c**	**<0.001**
**ICU admission, No. (%)**	**7 (54)b,d**	**35 (95)a,c**	**6 (40)b,d**	**37 (90)a,c**	**<0.001**

Abbreviations: ICU, intensive care unit; IQR, interquartile range; SD, standard deviation.

* P values were calculated either by the χ^2^ test or the Kruskal-Wallis test.

^a^ P<0.05 vs. placebo with β-lactam plus macrolide group.

^b^ P<0.05 vs. placebo with β-lactam plus fluoroquinolone group.

^c^ P<0.05 vs. methylprednisolone with β-lactam plus macrolide group.

^d^ P<0.05 vs. methylprednisolone with β-lactam plus fluoroquinolone group.

^e^ Patients could have more than one comorbidity.

^f^ Pneumonia severity index stratifies patients with CAP according to 30 day risk mortality of CAP in 5 different classes: risk classes from 1–3 (≤90 points) have a low mortality and risk classes 4 (91–130 points) and 5 (>130 points) have the highest mortality.

**Table 2:** Microbiologic identification for the intention-to-treat population.

	Placebo with β-lactams plus Macrolides Group (n = 13)	Placebo with β-lactams plus Fluoroquinolones Group (n = 37)	Methylprednisolone with β-lactams plus Macrolides Group (n = 15)	Methylprednisolone with β-lactams plus Fluoroquinolones Group (n = 41)	P value*
**Microorganism**					0.477
**Streptococcus pneumoniae**	2(15)	7(19)	3(20)	7(17)	0.986
**Legionella pneumophila**	0 (0)	1 (3)	1 (7)	2 (5)	0.777
**Gram-negative bacteria**	0 (0)	1 (3)	0 (0)	0 (0)	0.601
**Staphylococcus aureus**	0 (0)	0 (0)	0 (0)	1 (2)	0.663
**Coxiellaburnetti**	0 (0)	0 (0)	0 (0)	1 (2)	0.663
**Respiratory virus**	0 (0)	1 (3)	1 (7)	3 (7)	0.636
**Polymicrobial**	0 (0)	3 (8)	0 (0)	10 (24)	**0.018**
**Unknown etiology**	11 (85)^b^	24 (65)	10 (67)	17 (41)^a^	**0.022**

Data are shown as number of patients (%).

* P values were calculated by the χ^2^ test.

^a^ P<0.05 vs. placebo with β-lactams plus macrolides group.

^b^ P<0.05 vs. methylprednisolone with β-lactams plus fluoroquinolones group.

The most common antimicrobial treatment used was ceftriaxone plus levofloxacin in 33 [31%] patients in the group of placebo and 37 [34%] in the methylprednisolone group), ceftriaxone plus azithromycin was used in 11 (10%) patients of placebo group and 13 (12%) patients in the methylprednisolone group. More data about antimicrobial treatment, duration and time to the first dose are described elsewhere[12].

Treatment failure did not differ among the four groups (Table 3), nor did early treatment failure. However, late treatment failure did differ among the four groups (4 patients in the placebo with β-lactam plus macrolide group (31%) vs. 9 patients in the placebo with β-lactam plus fluoroquinolone group (24%) vs. 0 patients in the methylprednisolone with β-lactam plus macrolide group (0%) vs. 2 patients [5%] in the methylprednisolone with β-lactam plus fluoroquinolone group, overall crude comparison: p = 0.009). Similar results were obtained in the per-protocol population. Despite no statistically significant differences between groups were observed for in-hospital mortality in the intention-to-treat population, these differences were statistically significant in the per-protocol population (3 patients in the placebo with β-lactam plus macrolide group [23%] vs. 1 patients in the placebo with β-lactam plus fluoroquinolone group [3%] vs. 2 patients in the methylprednisolone with β-lactam plus macrolide group [14%] vs. 0 patients in the methylprednisolone with β-lactam plus fluoroquinolone group [0%], overall crude comparison p = 0.010). We did not find significant differences in treatment failure, early or late; or In-hospital mortality after adjusting for severity (PSI), year and centre of enrolmentin either the intention-to-treat population (Table 4) or the per-protocol population.

**Table 3:** Outcomes using descriptive statistics for the intention-to- treat population.

	Placebo with β-lactams plus Macrolides Group (n = 13)	Placebo with β-lactams plus Fluoroquinolones Group (n = 37)	Methylprednisolone with β-lactams plus Macrolides Group (n = 15)	Methylprednisolone with β-lactams plus Fluoroquinolones Group (n = 41)	P value*
**Primary Outcomes**					
**Treatment failure, No. (%)^d^**	4 (31)	10 (27)	2 (13)	6 (15)	0.374
**Early treatment failure (0–72 h), No. (%)^e^**	0 (0)	3 (8)	2 (13)	4 (10)	0.626
**Early mechanical ventilation, No. (%)**	0 (0)	3 (8)	1 (7)	3 (7)	0.780
**Early septic shock, No. (%)**	0 (0)	0 (0)	2 (13)	0 (0)	**0.006**
**Death within 0–72 h, No. (%)**	0 (0)	1 (3)	1 (7)	1 (2)	0.751
**Late treatment failure (72–120 h), No. (%)^e^**	4 (31)	9 (24)	0 (0)	2 (5)	**0.009**
**Radiographic progression, No. (%)**	2 (15)	5 (14)	0 (0)	1 (2)	0.122
**Respiratory failure, No. (%)**	2 (15)	2 (5)	0 (0)	1 (2)	0.208
**Late mechanical ventilation, No. (%)**	2 (15)	1 (3)	0 (0)	1 (2)	0.125
**Late septic shock, No. (%)**	1 (8)	3 (8)	0 (0)	0 (0)	0.198
**Death within 72–120 h, No. (%)**	0 (0)	0 (0)	0 (0)	0 (0)	-
**Secondary Outcomes**					
**C-reactive protein, mg/L**					
**Day 1 (n = 98)**					0.062
**Mean (SD)**	230 (80)	231 (67)	191 (95)	255 (84)	
**Median (IQR)**	244 (165; 294)	250 (182; 284)	238 (194; 244)	282 (204; 301)	
**Day 3 (n = 88)**					**0.023**
**Mean (SD)**	170 (97)	158 (80)	98 (67)	113 (66)	
**Median (IQR)**	190 (96; 241)	145 (102; 238)	105 (48; 119)	102 (67; 146)	
**Procalcitonin, ng/dL**					
**Day 1 (n = 97)**					**0.032**
**Mean (SD)**	6.8 (8.4)	6.3 (7.6)	2.4 (4.2)	4.8 (10.4)	
**Median (IQR)**	1.5 (0.5; 15.9)	4.2 (1.0; 8.4)	0.5 (0.1; 2.1)	1.3 (0.4; 4.1)	
**Day 3 (n = 88)**					**0.011**
**Mean (SD)**	4.2 (3.9)	3.1 (6.0)	0.9 (1.6)	1.6 (2.9)	
**Median (IQR)**	3.7 (0.4; 7.5)	1.0 (0.4; 2.9)	0.2 (0.1; 1.1)	0.5 (0.2; 1.7)	
**Interleukin-6, pg/dL**					
**Day 1 (n = 95)**					0.262
**Mean (SD)**	1042 (1367)	1165 (2588)	361 (446)	924 (2813)	
**Median (IQR)**	197 (169; 1534)	337 (219; 754)	165 (137; 243)	347 (134; 715)	
**Day 3 (n = 76)**					**<0.001**
**Mean (SD)**	250 (385)	278 (488)	70 (66)	103 (196)	
**Median (IQR)**	130 (102; 212)^c^	173 (109; 250)^c^	44 (14; 134)	49 (31; 67)^a^^,^^b^	
**Interleukin-8, pg/dL**					
**Day 1 (n = 88)**					0.405
**Mean (SD)**	141 (117)	4728 (24082)	102 (180)	249 (564)	
**Median (IQR)**	99 (57; 182)	76.5 (42; 151)	41 (36; 83)	80 (34.5; 161)	
**Day 3 (n = 78)**					0.206
**Mean (SD)**	203 (390)	134 (150)	47 (56)	139 (205)	
**Median (IQR)**	100 (36.5; 160)	67.5 (32; 194.5)	21.5 (17; 58)	53.5 (24.5; 149)	
**Interleukin-10, pg/dL**					
**Day 1 (n = 95)**					0.075
**Mean (SD)**	11.5 (11.3)	23.9 (55.8)	5.5 (7.2)	25.9 (85.6)	
**Median (IQR)**	7.8 (6.8; 11.5)	6.4 (3.5; 14.0)	4.1 (2.4; 4.8)	6.2 (2.9; 10.0)	
**Day 3 (n = 84)**					**0.019**
**Mean (SD)**	9.6 (11.7)	9.5 (11.8)	5.5 (8.9)	17.1 (77.7)	
**Median (IQR)**	6.9 (2.5; 10.0)	4.6 (2.7; 11.0)	4.0 (1.2; 4.6)	3.1 (1.1; 5.5)	
**Time to clinical stability, days^f^**					0.193
**Mean (SD)**	5.3 (4.2)	6.9 (5.7)	3.8 (1.9)	5.9 (4.8)	
**Median (IQR)**	4.0 (3.0; 6.0)	5.5 (3.5; 8.0)	4.0 (3.0; 4.0)	4.5 (3.0; 7.0)	
**Length of hospital stay, days**					0.920
**Mean (SD)**	12.9 (7.3)	16.4 (21.4)	12.4 (5.7)	15.9 (17.3)	
**Median (IQR)**	10.0 (8.0; 16.0)	12.0 (9.0; 15.0)	13.0 (8.0; 15.0)	11.0 (8.0; 14.5)	
**Length of ICU stay, days^g^**					0.833
**Mean (SD)**	7.0 (3.4)	7.9 (9.7)	5.8 (4.5)	8.6 (11.4)	
**Median (IQR)**	7.0 (4.0; 10.0)	6.0 (4.0; 8.0)	4.5 (4.0; 7.0)	5.0 (3.0; 8.0)	
**In-hospital mortality, No. (%)**	3 (23)	2 (5)	3 (20)	2 (5)	0.090

Abbreviations: ICU, intensive care unit; IQR, interquartile range; SD, standard deviation.

* P values were calculated either by the χ^2^ test or the Kruskal-Wallis test.

^a^ P<0.05 vs. placebo with β-lactams plus macrolides group.

^b^ P<0.05 vs. placebo with β-lactams plus fluoroquinolones group.

^c^ P<0.05 vs. methylprednisolone with β-lactams plus fluoroquinolones group.

^d^ Defined as the presence of early, late failure or both.

^e^ Several patients had more than 1 criteria of failure.

^f^ Clinical stability was considered to be attained when the following values were achieved for all parameters: temperature of 37.2°C or lower; heart rate of 100 beats/min or lower; systolic blood pressure of 90 mmHg or higher; and arterial oxygen tension of 60 mmHg or higher when the patient was not receiving supplemental oxygen. In patients who were receiving home oxygen therapy, stability was considered to be achieved when their oxygen needs were the same as before admission.

^g^ There were 7 patients in the placebo with β-lactam plus macrolide group, 34 patients in the placebo with β-lactams plus fluoroquinolones group, 6 patients in the methylprednisolone with β-lactams plus macrolides group, and 36 patients in the placebo with β-lactams plus fluoroquinolones group in the intention-to-treat population.

**Table 4:** Outcomes for the glucocorticosteroids and antibiotic combination treatments using logistic regression or cox proportional hazards models for the intention-to- treat population.

	OR or HR for the corticosteroid effect* (Placebo with β-lactams data were reference values) (95% CI)	OR or HR for the antibiotic effect* (Macrolides data were reference values) (95% CI)	OR or HR for the interaction effect* (95% CI)	P value for the corticosteroid effect^#^	P value for the antibiotic effect^#^	P value for the interaction effect^#^
**Primary Outcomes**						
**Treatment failure^a^**	0.37 (0.05 to 2.64)	1.30 (0.31 to 5.51)	1.30 (0.13 to 12.94)	0.320	0.722	0.824
**Early treatment failure (0–72 h)^b^**	NA^c^	NA^c^	NA^c^	>0.99	>0.99	>0.99
**Late treatment failure (72–120 h)^b^**	NA^c^	1.40 (0.31 to 6.36)	NA^c^	>0.99	0.663	>0.99
**Secondary Outcomes**						
**Time to clinical stability, days^d^**	1.65 (0.64 to 4.27)	0.58 (0.27 to 1.25)	0.63 (0.22 to 1.84)	0.297	0.163	0.403
**Length of hospital stay, days**	0.89 (0.16 to 4.85)	0.42 (0.07 to 2.71)	1.31 (0.10 to 17.58)	0.894	0.364	0.837
**Length of ICU stay, days^e^**	NA^c^	0.36 (0.05 to 2.71)	NA^c^	0.959	0.318	0.961
**In-hospital mortality**	0.72 (0.10 to 5.44)	0.34 (0.04 to 2.67)	1.48 (0.08 to 26.42)	0.769	0.307	0.791

Abbreviations: CI, confidence interval; HR, hazard ratio; NA; not available; OR, odds ratio.

*Estimate of the OR or HR comparing glucocorticosteroids and antibiotic combination treatments (placebo with β-lactams and macrolides being the reference groups) derived using either the logistic regression model or the Cox proportional hazards model adjusted for the severity (PSI score), year and centre of enrolment.

^#^ P values were calculated using either the logistic regression model or the Cox proportional hazards model adjusted for the severity (PSI score), year and centre of enrolment.

^a^ Defined as the presence of early, late failure or both.

^b^ Several patients had more than 1 criteria of failure.

^c^ Estimation failed due to numerical problem. Because the coefficients did not converge, no further models were fitted.

^d^ Clinical stability was considered to be attained when the following values were achieved for all parameters: temperature of 37.2°C or lower; heart rate of 100 beats/min or lower; systolic blood pressure of 90 mmHg or higher; and arterial oxygen tension of 60 mmHg or higher when the patient was not receiving supplemental oxygen. In patients who were receiving home oxygen therapy, stability was considered to be achieved when their oxygen needs were the same as before admission.

^e^ There were 7 patients in the placebo with β-lactam plus macrolide group, 34 patients in the placebo with β-lactams plus fluoroquinolones group, 6 patients in the methylprednisolone with β-lactams plus macrolides group, and 36 patients in the placebo with β-lactams plus fluoroquinolones group in the intention-to-treat population.

Results of statistical analyses of the inflammatory response at day 3, adjusting for the baseline inflammatory marker value, PSI risk class, year of recruitment, and centre of enrolment based on the ANCOVA models showed a significant effect of corticosteroids for IL-6, procalcitonin, and IL-8 (p<0.001, p = 0.016 and p = 0.028, respectively) (Table 5). For the interaction between glucocorticosteroids and antibiotics for procalcitonin a trend towards statistical significance was observed, mainly driven by lower values in the methylprednisolone with β-lactam plus macrolide group after adjustments for potential confounders. For IL-8, we observed lower values in the methylprednisolone with β-lactam plus macrolide group, although the interaction was not significant. Similar results were obtained in the per-protocol population.

**Table 5:** Inflammatory response on day 3 for the glucocorticosteroids and antibiotic combination treatments using ANCOVA models for the intention-to- treat population.

	Placebo with β-lactams plus Macrolides Group LS mean (95% CI)*	Placebo with β-lactams plus Fluoroquinolones Group LS mean (95% CI)*	Methylprednisolone with β-lactams plus Macrolides Group LS mean (95% CI)*	Methylprednisolone with β-lactams plus Fluoroquinolones Group LS mean (95% CI)*	P value for the corticosteroid effect^#^	P value for the antibiotic effect^#^	P value for the interaction effect^#^
**C-reactive protein at day 3, mg/L (n = 83)**	83.2 (43.3 to 160)	107.2 (61.6 to 186.8)	71.2 (35.2 to 144)	79.4 (47.1 to 133.8)	0.336	0.562	0.774
**Procalcitonin at day 3, ng/L (n = 85)**	1.10 (0.57 to 2.10)	0.89 (0.52 to 1.51)	0.39 (0.20 to 0.75)	0.78 (0.47 to 1.29)	**0.016**	0.421	0.066
**Interleukin-6 at day 3, pg/dL (n = 70)**	104.3 (50.5 to 215.5)	134.4 (71.4 to 252.9)	32.2 (13.6 to 76.6)	48 (26.3 to 87.9)	**<0.001**	0.374	0.804
**Interleukin-8 at day 3, pg/dL (n = 69)**	78.4 (30.2 to 203.8)	39.9 (17.1 to 92.8)	20.9 (7.4 to 59.1)	31.3 (14.3 to 68.8)	**0.028**	0.782	0.159
**Interleukin-10 at day 3, pg/dL (n = 79)**	4.92 (2.60 to 9.35)	5.52 (3.21 to 9.52)	4.34 (2.20 to 8.57)	2.72 (1.62 to 4.55)	0.076	0.571	0.232

Abbreviations: ANCOVA, analysis of covariance; CI, confidence interval; LS, least square.

* LS mean: least square mean for the inflammatory market at day 3 variables in the ANCOVA model.

^#^ P values were calculated using the ANCOVA models adjusted for the inflammatory marker at day 1 (baseline), severity (PSI score), year and centre of enrolment.

Analyses of primary and secondary outcomes, adjusting for age and ICU admission, showed no significance differences between groups (Tables B and C in S1 Supplementary file). The results of the methods for assessing the quality of the models are shown in Table D in S1 Supplementary file. Comparing only patients who received methylprednisolone with a β-lactam plus macrolide with the overall population, we did not observe significant differences (Tables E and F in S1 Supplementary file).

## Discussion

In this post-hoc exploratory analysis, we observed that patients who received glucocorticosteroids combined with macrolides did not have worse outcomes than those patients who received other combinations. Thus it does not appear to be unsafe to combine a macrolide with glucocorticosteroids when treating CAP, even though both have immune modulating effects. Furthermore, we observed a lower rate of late treatment failure and a trend of interaction for improved trend in biomarker findings, when using both glucocorticosteroids and macrolides, as reflected by procalcitonin at day 3.

Severe pneumonia patients have shown increased serum levels of IL-6, IL-8 and IL-10, and the excess of IL-6 and IL-10 are associated with an increase in mortality from 4.8% to 11.4%[26]. Corticosteroids and macrolides share some anti-inflammatory effects regulating cytokine release[27]. As regards macrolides, this effect has been observed in *in vitro* and *in vivo* studies. In addition, macrolides have effects on structural cells of the respiratory tract such as endothelial and epithelial cells. In animal studies macrolides show a reduction of histological inflammatory signs[28]. These studies have been performed predominantly with *Streptococcus pneumoniae* or *Mycoplasma pneumoniae* models of pneumonia. In an *in-vitro* study, the macrolides showed an improvement in sensitive corticosteroids and higher inhibition in IL-8 levels[29]. In a recent human study, treatment with corticosteroids and macrolides reduced the level of inflammatory biomarkers such as IL-6 and IL-8 in bronchoalveolar lavage of patients with non-responding pneumonia[30], also in a recent post-hoc analysis of STEP study the patients who receiving corticosteroids plus macrolides presented lower re-hospitalisation than those received β-lactam monotherapy[31]. Macrolides and corticosteroids act on different targets[32], and their combination could potentiate their immunomodulatory effects. Interestingly, quinolones have also been associated with immunomodulatory effects during in vitro experiments, ex-vivo investigations, in vivo pre-clinical models and in the clinical setting[33,34].

Treatment failure is common in patients with severe pneumonia, reaching rates of 35% in high-risk patients (PSI class V)[35,36]. This rate was reduced with corticosteroid treatment to 13% in the original trial[12] and, in this post-hoc analysis, we found an even lower rate in patients receiving antibiotic treatment with a macrolide and a ß-lactam, along with the glucocorticosteroids, where late treatment failure was reduced to 0%. However, because we had few events, we were unpowered to observe a potential statistically significant interaction between glucocorticosteroid and antibiotic groups.

Our study included a specific population of patients with community-acquired pneumonia with high inflammatory response measured by high CRP levels at randomization, and all patients received β-lactam antibiotic treatment. These criteria allowed us to analyse the target effect of macrolides and glucocorticosteroids, taking account that different cytokine profiles were described accordingly to pneumonia aetiology, severity and due to the bactericidal properties of antimicrobial treatments[26]. There are more than one definition for treatment failure for community-acquired pneumonia, and we choose the definition developed in the Neumofail study, which has shown relationship with mortality[36].

Our study has limitations that must be acknowledged. First, the antibiotic choice was not randomized, resulting in heterogeneous groups with differences in baseline characteristics including severity and admission to ICU. It would appear that attending physicians treat elderly patients with comorbidities with macrolides and patients admitted to ICU with fluoroquinolones. We tried to overcome this limitation by adjusting for PSI score, which accounts for age, comorbidities and severity. Second, the number of patients who received macrolides was small, hindering the possibility of a fully-adjusted analysis for proper comparison with patients who received fluoroquinolones and their interaction with glucocorticosteroids. Third, we did not measure baseline cortisol, which may be an important marker for measuring the effects of glucocorticosteroids.

In conclusion the glucocorticosteroids and macrolides combination had no statistically significant association with main clinical outcomes compared with other combinations in patients with severe community acquired pneumonia and a high inflammatory response after taking account potential confounders. We believe this report could be a hypothesis generator to further RCTs combining glucocorticosteroids with macrolides or fluoroquinolones.

## Supporting information

S1 Supplementary file (DOCX)

S1 Database (SAV)
