# Supplementary material for: Treatment with macrolides and glucocorticosteroids in severe community-acquired pneumonia: A post-hoc exploratory analysis of a randomized controlled trial
Source: PLoS One. 2017 Jun 15;12(6):e0178022. doi: 10.1371/journal.pone.0178022 (PMC5472276; doi:10.1371/journal.pone.0178022)
Supplement: S1 Supplementary file — (DOCX) [file pone.0178022.s001.docx]

**Supplementary Online Content**

**Treatment with Macrolides and Glucocorticosteroids in Severe Community-Acquired Pneumonia: a post-hoc exploratory analysis of a randomized controlled trial**

Adrian Ceccato^1, 2^; Catia Cilloniz^1^; Otavio T. Ranzani^1,3^; Rosario Menendez^4^; Carles Agusti^1^; Albert Gabarrus^1^; Miquel Ferrer^1^; Oriol Sibila^5^; Michael S Niederman^6^; Antoni Torres*^1^.

^1^ Department of Pneumology, Institut Clinic de Respiratori, Hospital Clinic of Barcelona - Institut d'Investigacions Biomèdiques August Pi i Sunyer (IDIBAPS), University of Barcelona (UB) - SGR 911 - Ciber de Enfermedades Respiratorias (Ciberes, CB06/06/0028) Villarroel 170 (08036) Barcelona, Spain.

^2^ Seccion Neumologia, Hospital Nacional Prof. Alejandro Posadas, Illia y Marconi s/n (1684) Palomar, Argentina.

^3^ Respiratory Intensive Care Unit, Pulmonary Division, Heart Institute, Hospital das Clínicas, University of São Paulo, Av. Dr. Arnaldo, 455-Cerqueira César - CEP: 01246903 São Paulo, Brazil.

^4^ Servicio de Neumología, IIS/Hospital Universitario y Politécnico La Fe, Avinguda de Fernando Abril Martorell, nº 106 (46026) Valencia, CIBERES, Spain.

^5^Servei de Pneumologia, Hospital de la Santa Creu i Sant Pau, Sant Antoni Maria Claret 167 (08025) Barcelona, Spain.

^6^ Division of Pulmonary and Critical Care Medicine, Weill Cornell Medical College, New York Presbyterian/Weill Cornell Medical Center, 425 East 61st St, NY10065 New York, NY.

Clinicaltrials.gov Identifier: NCT00908713

***Corresponding Author**: Prof. Antoni Torres

Department of Pneumology, Hospital Clinic of Barcelona . Villarroel 170 (08036) Barcelona, Spain.

Phone +34932275549 Fax +34932275549

Email: [atorres@clinic.ub.es](mailto:atorres@clinic.ub.es)

**eMethods**

**Ethics committee and clinical trials registration**

The study was approved by the Ethic Committee of the Hospital Clinic of Barcelona (Barcelona, Spain; Register: 2003/1421) at January 7^th^ of 2003. Patients were prospectively enrolled and followed up from June 2004 through February 2012.

The study started in 2004 but the protocol was not submitted to [clinicaltrials.gov](http://clinicaltrials.gov/) until 2009. The reason was that in 2003 registration in [clinicaltrials.gov](http://clinicaltrials.gov/) was not required for local ethics committees. Until the study reached 60 patients enrolled and the interim analysis could be performed, the protocol was not registered in [clinicaltrials.gov](http://clinicaltrials.gov/) (in May 2009). The interim analysis was performed after registration.

The authors confirm that all ongoing and related trials for this drug/intervention are registered.

**Statistical Analysis**

Additionally we performed logistic regression models to examine differences in the rate of treatment failure between the groups, as well as in early and late treatment failure and in-hospital mortality, adjusting for the age, intensive care admission, year of recruitment, and centre. Time to clinical stability, length of ICU and hospital stay between the groups were also analyzed by means of Cox proportional hazards models, adjusting for the age, intensive care admission, year of recruitment, and centre. The odds ratio (OR) or hazard ratio (HR) and their 95% confidence intervals (CI) were estimated. Similarly, we fit analysis of covariance (ANCOVA) models to analyze the inflammatory response at day 3, adjusting for the baseline inflammatory marker value, age, intensive care admission, year of recruitment, and centre. Inflammatory markers were log-transformed to fit the ANCOVA model. Each treatment effect was estimated by the least squares mean and its 95% CI. The quality of the logistic regression models and ANCOVA models were tested using the Hosmer-Lemeshow test and Akaike information criterion, respectively. All tests were 2-tailed and significance was set at 0.05. All analyses were performed with IBM SPSS Statistics version 22.0 (Armonk, New York, USA).

Table A. Baseline Characteristics

| **Variable** | **Excluded Patients from the Analysis**  **(n=14)** | **Included Patients in the Analysis**  **(n=106)** | **P value*** |
| --- | --- | --- | --- |
| **Age, years Mean (SD) Median (IQR)** | 81.6 (7.7) 82.0 (78.0; 88.0) | 63.1 (19.6) 66.0 (50.0; 81.0) | **<0.001** |
| **Male sex, No. (%)** | 11 (79) | 63 (59) | 0.166 |
| **Current smoker, No. (%)** | 2 (14) | 30 (28) | 0.349 |
| **Pre-existing comorbid conditions, No. (%)^a^** |  |  |  |
| **Diabetes mellitus** | 3 (21) | 20 (19) | 0.731 |
| **Chronic pulmonary disease** | 3 (21) | 16 (15) | 0.464 |
| **Congestive heart failure** | 6 (43) | 40 (38) | 0.774 |
| **History of malignancy** | 3 (21) | 8 (8) | 0.119 |
| **Ischemic heart disease** | 3 (21) | 18 (17) | 0.710 |
| **Pneumonia Severity Index score Mean (SD) Median (IQR)** | 140.4 (26.1) 141.1 (132.0; 163.0) | 104.3 (35.5) 106.0 (83.0; 131.0) | **<0.001** |
| **Risk class, No. (%)^b^** |  |  | **0.001** |
| **I-III** | 1 (7) | 31 (29) | 0.109 |
| **IV** | 2 (14) | 45 (43) | **0.042** |
| **V** | 11 (79) | 30 (28) | **<0.001** |
| **ICU admission, No. (%)** | 5 (36) | 85 (80) | **<0.001** |

Abbreviations: ICU, intensive care unit; IQR, interquartile range; SD, standard deviation.

* P values were calculated either by the χ^2^ test, the Fisher exact test or the Mann-Whitney test.

^a^ Patients could have more than one comorbidity.

^b^ Pneumonia severity index stratifies patients with CAP according to 30 day risk mortality of CAP in 5 different classes: risk classes from 1-3 (≤90 points) have a low mortality and risk classes 4 (91-130 points) and 5 (>130 points) have the highest mortality.

Table B. Outcomes for the glucocorticosteroids and antibiotic combination treatments using Logistic Regression or Cox Proportional Hazards Models for the Intention-to- Treat Population (Adjusted for Age. ICU admission, year and centre of enrolment)

|  | **OR or HR for the corticosteroid effect* (Placebo with β-lactams data were reference values) (95% CI)** | **OR or HR for the antibiotic effect* (Macrolides data were reference values) (95% CI)** | **OR or HR for the interaction effect* (95% CI)** | **P value for the corticosteroid effect^#^** | **P value for the antibiotic effect^#^** | **P value for the interaction effect^#^** |
| --- | --- | --- | --- | --- | --- | --- |
| **Primary Outcomes** |  |  |  |  |  |  |
| **Treatment failure^a^** | 0.43 (0.06 to 3.06) | 0.54 (0.11 to 2.62) | 1.08 (0.11 to 10.56) | 0.403 | 0.445 | 0.950 |
| **Early treatment failure (0-72 h)^b^** | NA^c^ | NA^c^ | NA^c^ | >0.99 | >0.99 | >0.99 |
| **Late treatment failure (72-120 h)^b^** | NA^c^ | 0.53 (0.09 to 3.22) | NA^c^ | >0.99 | 0.486 | >0.99 |
| **Secondary Outcomes** |  |  |  |  |  |  |
| **Time to clinical stability, median (IQR), days^d^** | 1.65 (0.64 to 4.26) | 0.77 (0.34 to 1.72) | 0.67 (0.24 to 1.93) | 0.297 | 0.523 | 0.464 |
| **Length of hospital stay, median (IQR), days** | 1.02 (0.19 to 5.42) | 0.30 (0.04 to 2.29) | 0.92 (0.07 to 12.47) | 0.984 | 0.246 | 0.544 |
| **Length of ICU stay, median (IQR), days^e^** | NA^c^ | 0.27 (0.03 to 2.18) | NA^c^ | 0.959 | 0.218 | 0.963 |
| **In-hospital mortality** | 0.95 (0.13 to 6.67) | 0.23 (0.03 to 2.03) | 1.00 (0.06 to 17.43) | 0.957 | 0.183 | >0.99 |

Abbreviations: CI, confidence interval; HR, hazard ratio; NA; not available; OR, odds ratio.

*Estimate of the OR or HR comparing glucocorticosteroids and antibiotic combination treatments (placebo with β-lactams and macrolides being the reference groups) derived using either the logistic regression model or the Cox proportional hazards model adjusted for the age, intensive care admission, year and centre of enrolment.

^#^ P values were calculated using either the logistic regression model or the Cox proportional hazards model adjusted for the age, intensive care admission, year and centre of enrolment.

^a^ Defined as the presence of early, late failure or both.

^b^ Several patients had more than 1 criteria of failure.

^c^ Estimation failed due to numerical problem. Because the coefficients did not converge, no further models were fitted.

^d^ Clinical stability was considered to be attained when the following values were achieved for all parameters: temperature of 37.2 °C or lower; heart rate of 100 beats/min or lower; systolic blood pressure of 90 mmHg or higher; and arterial oxygen tension of 60 mmHg or higher when the patient was not receiving supplemental oxygen. In patients who were receiving home oxygen therapy, stability was considered to be achieved when their oxygen needs were the same as before admission.

^e^ There were 7 patients in the placebo with β-lactam plus macrolide group, 34 patients in the placebo with β-lactams plus fluoroquinolones group, 6 patients in the methylprednisolone with β-lactams plus macrolides group, and 36 patients in the placebo with β-lactams plus fluoroquinolones group in the intention-to-treat population.

Table C. Inflammatory Response on Day 3 for the Glucocorticosteroids and Antibiotic Combination Treatments Using ANCOVA Models for the Intention-to- Treat Population (Adjusted for Baseline level, Age. ICU admission, year and centre of enrolment)

|  | **Placebo with β-lactams plus Macrolides Group**  **LS mean (95% CI)*** | **Placebo with β-lactams plus Fluoroquinolones Group**  **LS mean (95% CI)*** | **Methylprednisolone with β-lactams plus Macrolides Group**  **LS mean (95% CI)*** | **Methylprednisolone with β-lactams plus Fluoroquinolones Group**  **LS mean (95% CI)*** | **P value for the corticosteroid effect^#^** | **P value for the antibiotic effect^#^** | **P value for the interaction effect^#^** |
| --- | --- | --- | --- | --- | --- | --- | --- |
| **C-reactive protein at day 3, mg/L (n=83)** | 91.7 (44.4 to 189.5) | 130.8 (70.6 to 242.5) | 89.2 (42.2 to 188.7) | 96.4 (57.7 to 176.4) | 0.433 | 0.445 | 0.536 |
| **Procalcitonin at day 3, ng/L (n=85)** | 1.30 (0.58 to 2.96) | 1.00 (0.51 to 1.97) | 0.55 (0.25 to 1.24) | 0.87 (0.45 to 1.68) | **0.039** | 0.768 | 0.145 |
| **Interleukin-6 at day 3, pg/dL (n=70)** | 190.2 (66.7 to 542.1) | 197.9 (78.4 to 499.2) | 52.5 (16.5 to 167) | 71 (26.5 to 190.2) | **<0.001** | 0.659 | 0.665 |
| **Interleukin-8 at day 3, pg/dL (n=69)** | 123 (31.7 to 476.6) | 60 (17.5 to 206.5) | 34 (9 to 128.2) | 45.6 (15 to 138.6) | **0.029** | 0.666 | 0.175 |
| **Interleukin-10 at day 3, pg/dL (n=79)** | 4.56 (2.08 to 9.99) | 4.97 (2.55 to 9.69) | 3.89 (1.72 to 8.80) | 2.34 (1.20 to 4.54) | 0.054 | 0.508 | 0.218 |

Abbreviations: ANCOVA, analysis of covariance; CI, confidence interval; LS, least square.

* LS mean: least square mean for the inflammatory market at day 3 variables in the ANCOVA model.

^#^ P values were calculated using the ANCOVA models adjusted for the inflammatory marker at day 1 (baseline), age, intensive care admission, year and centre of enrolment.

Table D. Quality of Each Logistic Regression Model or ANCOVA Model Using the Hosmer-Lemeshow Test or Akaike's Information Criterion for Outcomes

|  | **Hosmer–Lemeshow Test** | | **Akaike's Information Criterion** | |
| --- | --- | --- | --- | --- |
|  | **P value*** | **P value^#^** | **Value^$^** | **Value^&^** |
| **Primary Outcomes** |  |  |  |  |
| **Treatment failure** | 0.618 | 0.539 | - | - |
| **Early treatment failure (0-72 h)** | 0.872 | 0.496 | - | - |
| **Late treatment failure (72-120 h)** | 0.986 | 0.612 | - | - |
| **Secondary Outcomes** |  |  |  |  |
| **C-reactive protein at day 3** | - | - | 220.292 | 211.485 |
| **Procalcitonin at day 3** | - | - | 214.810 | 224.648 |
| **Interleukin-6 at day 3** | - | - | 181.105 | 190.016 |
| **Interleukin-8 at day 3** | - | - | 204.825 | 208.907 |
| **Interleukin-10 at day 3** | - | - | 196.011 | 201.982 |
| **In-hospital mortality** | 0.820 | 0.572 | - | - |

Abbreviations: ANCOVA, analysis of covariance.

* P values were calculated using the logistic regression model adjusted for the severity (PSI score), year and centre of enrolment.

^#^ P values were calculated using the logistic regression model adjusted for the age, intensive care admission, year and centre of enrolment.

^$^ Values were calculated using the ANCOVA models adjusted for the inflammatory marker at day 1 (baseline), severity (PSI score), year and centre of enrolment.

^&^ Values were calculated using the ANCOVA models adjusted for the inflammatory marker at day 1 (baseline), age, intensive care admission, year and centre of enrolment.

Table E. Baseline Characteristics

| **Variable** | **Methylprednisolone with β-lactams plus Macrolides Group**  **(n=15)** | **Other Groups**  **(n=91)** | **P value*** |
| --- | --- | --- | --- |
| **Age, years Mean (SD) Median (IQR)** | 78.5 (10.7) 82.0 (78.0; 84.0) | 60.5 (19.6) 62.0 (47.0; 77.0) | **<0.001** |
| **Male sex, No. (%)** | 8 (53) | 55 (60) | 0.604 |
| **Current smoker, No. (%)** | 0 (0) | 30 (33) | **0.005** |
| **Pre-existing comorbid conditions, No. (%)^a^** |  |  |  |
| **Diabetes mellitus** | 5 (33) | 15 (16) | 0.153 |
| **Chronic pulmonary disease** | 2 (13) | 14 (15) | >0.99 |
| **Congestive heart failure** | 11 (73) | 29 (32) | **0.002** |
| **History of malignancy** | 1 (7) | 7 (8) | >0.99 |
| **Ischemic heart disease** | 7 (47) | 11 (12) | **0.004** |
| **Pneumonia Severity Index score Mean (SD) Median (IQR)** | 133.5 (35.5) 134.0 (130.0; 149.0) | 99.3 (33.2) 102.0 (79.0; 123.0) | **<0.001** |
| **Risk class, No. (%)^b^** |  |  | **<0.001** |
| **I-III** | 1 (7) | 30 (33) | 0.062 |
| **IV** | 3 (20) | 42 (46) | 0.058 |
| **V** | 11 (73) | 19 (21) | **<0.001** |
| **ICU admission, No. (%)** | 6 (40) | 79 (87) | **<0.001** |

Abbreviations: ICU, intensive care unit; IQR, interquartile range; SD, standard deviation.

* P values were calculated either by the χ^2^ test, the Fisher exact test or the Mann-Whitney test.

^a^ Patients could have more than one comorbidity.

^b^ Pneumonia severity index stratifies patients with CAP according to 30 day risk mortality of CAP in 5 different classes: risk classes from 1-3 (≤90 points) have a low mortality and risk classes 4 (91-130 points) and 5 (>130 points) have the highest mortality.

Table F. Outcomes Using Descriptive Statistics for the Intention-to- Treat Population

|  | **Methylprednisolone with β-lactams plus Macrolides Group**  **(n=15)** | **Other Groups**  **(n=91)** | **P value*** |
| --- | --- | --- | --- |
| **Primary Outcomes** |  |  |  |
| **Treatment failure, No. (%)^a^** | 2 (13) | 20 (22) | 0.732 |
| **Early treatment failure (0-72 h), No. (%)^b^** | 2 (13) | 7 (8) | 0.612 |
| **Early mechanical ventilation, No. (%)** | 1 (7) | 6 (7) | >0.99 |
| **Early septic shock, No. (%)** | 2 (13) | 0 (0) | **0.019** |
| **Death within 0-72 h, No. (%)** | 1 (7) | 2 (2) | 0.370 |
| **Late treatment failure (72-120 h), No. (%)^f^** | 0 (0) | 15 (16) | 0.121 |
| **Radiographic progression, No. (%)** | 0 (0) | 8 (9) | 0.597 |
| **Respiratory failure, No. (%)** | 0 (0) | 5 (5) | >0.99 |
| **Late mechanical ventilation, No. (%)** | 0 (0) | 4 (4) | >0.99 |
| **Late septic shock, No. (%)** | 0 (0) | 4 (4) | >0.99 |
| **Death within 72-120 h, No. (%)** | 0 (0) | 0 (0) | - |
| **Secondary Outcomes** |  |  |  |
| **C-reactive protein, mg/L** |  |  |  |
| **Day 1 (n=98) Mean (SD) Median (IQR)** | 191 (95) 238 (194; 244) | 6.3 (7.6) 4.2 (1.0; 8.4) | **0.037** |
| **Day 3 (n=88) Mean (SD) Median (IQR)** | 98 (67) 105 (48; 119) | 139 (79) 122 (79; 208) | 0.103 |
| **Procalcitonin, ng/dL** |  |  |  |
| **Day 1 (n=97) Mean (SD) Median (IQR)** | 2.4 (4.2) 0.5 (0.1; 2.1) | 5.6 (9.0) 1.9 (0.5; 7.1) | **0.024** |
| **Day 3 (n=88) Mean (SD) Median (IQR)** | 0.9 (1.6) 0.2 (0.1; 1.1) | 2.6 (4.5) 0.7 (0.3; 2.9) | **0.013** |
| **Interleukin-6, pg/dL** |  |  |  |
| **Day 1 (n=95) Mean (SD) Median (IQR)** | 361 (446) 165 (137; 243) | 1035 (2538) 329 (175; 774) | 0.078 |
| **Day 3 (n=76) Mean (SD) Median (IQR)** | 70 (66) 44 (14; 134) | 197 (372) 106 (46; 209) | 0.078 |
| **Interleukin-8, pg/dL** |  |  |  |
| **Day 1 (n=88) Mean (SD) Median (IQR)** | 102 (180) 41 (36; 83) | 1906 (14716) 86 (36; 159) | 0.140 |
| **Day 3 (n=78) Mean (SD) Median (IQR)** | 47 (56) 21.5 (17; 58) | 148 (230) 66 (24.5; 161.5) | **0.045** |
| **Interleukin-10, pg/dL** |  |  |  |
| **Day 1 (n=95) Mean (SD) Median (IQR)** | 5.5 (7.2) 4.1 (2.4; 4.8) | 23.0 (67.9) 7.2 (3.0; 12.0) | **0.027** |
| **Day 3 (n=84) Mean (SD) Median (IQR)** | 5.5 (8.9) 4.0 (1.2; 4.6) | 13.1 (53.4) 3.9 (2.1; 8.3) | 0.304 |
| **Time to clinical stability, days^c^ Mean (SD) Median (IQR)** | 3.8 (1.9) 4.0 (3.0; 4.0) | 6.3 (5.1) 5.0 (3.0; 8.0) | 0.079 |
| **Length of hospital stay, days Mean (SD) Median (IQR)** | 12.4 (5.7) 13.0 (8.0; 15.0) | 15.7 (18.0) 11.0 (8.0; 15.0) | 0.875 |
| **Length of ICU stay, days^d^ Mean (SD) Median (IQR)** | 5.8 (4.5) 4.5 (4.0; 7.0) | 8.2 (10.1) 6.0 (4.0; 8.0) | 0.542 |
| **In-hospital mortality, No. (%)** | 3 (20) | 7 (8) | 0.149 |

Abbreviations: ICU, intensive care unit; IQR, interquartile range; SD, standard deviation.

* P values were calculated either by the χ^2^ test or the Mann-Whitney test.

^a^ Defined as the presence of early, late failure or both.

^b^ Several patients had more than 1 criteria of failure.

^c^ Clinical stability was considered to be attained when the following values were achieved for all parameters: temperature of 37.2 °C or lower; heart rate of 100 beats/min or lower; systolic blood pressure of 90 mmHg or higher; and arterial oxygen tension of 60 mmHg or higher when the patient was not receiving supplemental oxygen. In patients who were receiving home oxygen therapy, stability was considered to be achieved when their oxygen needs were the same as before admission.

^d^ There were 6 patients in the methylprednisolone with β-lactams plus macrolides group, and 77 patients in the other groups in the intention-to-treat population.

STROBE checklist.

|  | Item No | | Recommendation | Page |
| --- | --- | --- | --- | --- |
| **Title and abstract** | | 1 | (*a*) Indicate the study’s design with a commonly used term in the title or the abstract | 1 |
|  |  |  | (*b*) Provide in the abstract an informative and balanced summary of what was done and what was found | 2 |
| Introduction | | | |  |
| Background/rationale | | 2 | Explain the scientific background and rationale for the investigation being reported | 3 |
| Objectives | | 3 | State specific objectives, including any prespecified hypotheses | 5 |
| Methods | | | |  |
| Study design | | 4 | Present key elements of study design early in the paper | 6 |
| Setting | | 5 | Describe the setting, locations, and relevant dates, including periods of recruitment, exposure, follow-up, and data collection | 6 |
| Participants | | 6 | (*a*) *Cohort study*—Give the eligibility criteria, and the sources and methods of selection of participants. Describe methods of follow-up  *Case-control study*—Give the eligibility criteria, and the sources and methods of case ascertainment and control selection. Give the rationale for the choice of cases and controls  *Cross-sectional study*—Give the eligibility criteria, and the sources and methods of selection of participants | 6 |
|  |  |  | (*b*) *Cohort study*—For matched studies, give matching criteria and number of exposed and unexposed  *Case-control study*—For matched studies, give matching criteria and the number of controls per case |  |
| Variables | | 7 | Clearly define all outcomes, exposures, predictors, potential confounders, and effect modifiers. Give diagnostic criteria, if applicable |  |
| Data sources/ measurement | | 8* | For each variable of interest, give sources of data and details of methods of assessment (measurement). Describe comparability of assessment methods if there is more than one group | *6, 7* |
| Bias | | 9 | Describe any efforts to address potential sources of bias | 7, 8 |
| Study size | | 10 | Explain how the study size was arrived at | 6 |
| Quantitative variables | | 11 | Explain how quantitative variables were handled in the analyses. If applicable, describe which groupings were chosen and why | 8 |
| Statistical methods | | 12 | (*a*) Describe all statistical methods, including those used to control for confounding | 8, 9 |
|  |  |  | (*b*) Describe any methods used to examine subgroups and interactions |  |
|  |  |  | (*c*) Explain how missing data were addressed |  |
|  |  |  | (*d*) *Cohort study*—If applicable, explain how loss to follow-up was addressed  *Case-control study*—If applicable, explain how matching of cases and controls was addressed  *Cross-sectional study*—If applicable, describe analytical methods taking account of sampling strategy |  |
|  |  |  | (*e*) Describe any sensitivity analyses |  |
| Results | |  |  |  |
| Participants | | | |  |
| Participants  Descriptive data | 13*  14* | | (b) Give reasons for non-participation at each stage | 10 |
|  |  |  | (c) Consider use of a flow diagram | Fig 1 |
|  |  |  | (a) Give characteristics of study participants (eg demographic, clinical, social) and information on exposures and potential confounders | Table 1, 2 |
| Descriptive data  Outcome data | 14*  15* | | (b) Indicate number of participants with missing data for each variable of interest | Table 1 |
|  |  |  | (c) *Cohort study*—Summarise follow-up time (eg, average and total amount) |  |
|  |  |  | *Cohort study*—Report numbers of outcome events or summary measures over time | *Table 3* |
| Outcome data  Main results | 15*  16 | | *Case-control study—*Report numbers in each exposure category, or summary measures of exposure |  |
|  |  |  | *Cross-sectional study—*Report numbers of outcome events or summary measures |  |
|  |  |  | (*a*) Give unadjusted estimates and, if applicable, confounder-adjusted estimates and their precision (eg, 95% confidence interval). Make clear which confounders were adjusted for and why they were included | *Table 3* |
| Main results  Other analyses | 16  17 | | (*b*) Report category boundaries when continuous variables were categorized |  |
|  |  |  | (*c*) If relevant, consider translating estimates of relative risk into absolute risk for a meaningful time period |  |
|  |  |  | Report other analyses done—eg analyses of subgroups and interactions, and sensitivity analyses | Table 4, eTable1-4 |
| Discussion |  | | Report other analyses done—eg analyses of subgroups and interactions, and sensitivity analyses | Table 4, eTable1-4 |
| Key results | | | |  |
| Limitations | 19 | | Discuss limitations of the study, taking into account sources of potential bias or imprecision. Discuss both direction and magnitude of any potential bias | 14 |
| Interpretation | 20 | | Give a cautious overall interpretation of results considering objectives, limitations, multiplicity of analyses, results from similar studies, and other relevant evidence | 14 |
| Generalisability | 21 | | Discuss the generalisability (external validity) of the study results | 14 |
| Other information |  | | Discuss the generalisability (external validity) of the study results | 14 |
| Funding | | | |  |
| Funding | 22 | | Give the source of funding and the role of the funders for the present study and, if applicable, for the original study on which the present article is based | 15 |
